# Ozone as a Catalyst for Alzheimer’s Disease and Sleep Disorders: Implications for Neurodegeneration Induced by Air Pollution

**DOI:** 10.3390/biology15201904

**Published:** 2026-10-21

**Authors:** Alejandro López-Landa, Emiliano González-Sánchez, Javier Pérez-Villavicencio, Norma Serrano García, Carlos García-Hernández, Ángel Lee, Moisés Rubio-Osornio, Carmen Rubio

**Affiliations:** 1 Faculty of Medicine, Meritorious Autonomous University of Puebla, Puebla 72570, Mexico; 2 Faculty of Medicine, Autonomous University of Chiapas, Chiapas 29050, Mexico; emiliano.gonzalez04@unach.mx; 3 Neurophysiology Department, National Institute of Neurology and Neurosurgery, Mexico City 14269, Mexiconorma.serrano@innn.edu.mx (N.S.G.); 4 Biological and Health Sciences Division, Metropolitan Autonomous University, Iztapalapa Campus, Mexico City 09340, Mexico; 5 Faculty of Medicine and Surgery, Southeastern Regional University, Oaxaca 68150, Mexico; 6 Public Health Intelligence Unit, National Institute of Public Health, Cuernavaca 62100, Mexico; 7 Department of Neurochemistry, National Institute of Neurology and Neurosurgery, Mexico City 14269, Mexico; moises.rubio@innn.edu.mx

**Keywords:** ozone, air pollution, Alzheimer’s disease, sleep disorders, neuroinflammation, oxidative stress

## Abstract

Tropospheric ozone, a highly reactive air pollutant, has emerged as a potential environmental risk factor for Alzheimer’s disease (AD) and its associated sleep disturbances, yet a comprehensive synthesis of the underlying mechanisms has remained limited. This narrative review summarizes experimental and epidemiological evidence on ozone exposure, neurodegeneration and disrupted sleep, organized around one working hypothesis: ozone generates a systemic oxidative–inflammatory signal that reaches the central nervous system, with sleep/circadian disruption and AD-related pathology as downstream outcomes that may reinforce each other. In experimental models, ozone is associated with oxidative stress, mitochondrial dysfunction, blood–brain barrier disruption, glial activation and neuroinflammation, overlapping with β-amyloid accumulation and tau hyperphosphorylation, and with reduced REM sleep and altered neurotransmitters; clock-gene changes have been shown mainly in peripheral tissue. Circadian disruption is a candidate effector, and current data cannot distinguish mediation from co-occurrence. Epidemiological evidence for ozone-specific associations with cognitive decline remains inconsistent at the population level compared with other pollutants such as PM_2.5_ or NO_2_, although some cohorts report pollutant-specific effects and modulation by the apolipoprotein E ε4 (APOE4) genotype. Most experimental studies use concentrations well above regulatory ambient standards. Overall, ambient ozone is a biologically plausible, but not yet epidemiologically confirmed, candidate risk factor for AD and sleep disorders. Future work should prioritize exposure-graded longitudinal studies and clarification of the dose–response relationship, including the effects of controlled medical ozone.

## Simple Summary

Ozone, an invisible gas found in smoggy air, may be an overlooked contributor to brain health amid growing concern about air pollution more broadly. This review brings together laboratory and population evidence on how ozone exposure could affect the brain and disrupt sleep, two factors increasingly linked to Alzheimer’s disease. Laboratory studies suggest that ozone can trigger inflammation and oxidative damage that may reach the brain, and may disturb sleep and the body’s internal clock. Poor sleep is known to impair the brain’s clearance of proteins linked to Alzheimer’s disease, but it has not been shown that this is how ozone acts. Most of this evidence comes from animal studies using ozone levels far higher than those typically found in city air, and population studies in humans have shown mixed results, with weaker and less consistent links than those found for other pollutants such as fine particulate matter. We therefore propose, as a hypothesis, that ozone triggers a body-wide inflammatory and oxidative signal that affects both sleep and Alzheimer’s-related changes. Taken together, the evidence positions ozone as a biologically plausible, though not yet epidemiologically confirmed, contributor to Alzheimer’s disease and sleep disruption.

## 1. Introduction

Alzheimer’s disease (AD) is a progressive neurodegenerative disorder characterized by cognitive decline and hallmark neuropathological features, including β-amyloid (Aβ) plaque accumulation, hyperphosphorylated tau neurofibrillary tangles, oxidative stress, neuroinflammation, and synaptic dysfunction [1,2,3,4]. Beyond cognitive deterioration, sleep disturbances represent one of the most frequent and impactful non-cognitive manifestations of AD, encompassing insomnia, sleep fragmentation, reduced slow-wave sleep, and alterations in rapid eye movement (REM) and circadian rhythms. These disruptions not only impair quality of life but also appear to accelerate neurodegenerative processes by promoting oxidative damage, neuroinflammation, and impaired clearance of Aβ [5,6,7]. Sleep and circadian regulation are intimately linked to brain homeostasis, involving a complex network of neural structures. The preoptic area and basal forebrain primarily regulate non-rapid eye movement (NREM) sleep, while REM sleep depends on the coordinated activity of the pedunculopontine nucleus, dorsolateral tegmentum, dorsal raphe nucleus, and locus coeruleus [8,9,10]. At the system’s core lies the suprachiasmatic nucleus (SCN), the brain’s master circadian pacemaker, which synchronizes the sleep–wake cycle with metabolic and molecular processes essential for neuronal integrity and memory consolidation [8,9,10]. Recent proposals suggest that environmental factors, especially atmospheric pollutants like tropospheric ozone (O_3_), may adversely affect sleep quality and the advancement of neurodegenerative disorders such as AD [11,12,13]. Ozone is naturally produced in the stratosphere through ultraviolet radiation acting on oxygen molecules; however, its accumulation in the troposphere as a secondary pollutant, resulting from photochemical reactions between nitrogen oxides (NOx) and volatile organic compounds from anthropogenic sources, poses an increasing health risk [14,15,16].

Experimental studies indicate that tropospheric ozone can provoke oxidative stress, neuroglial inflammation, mitochondrial dysfunction, synaptic changes, and impairment of the blood–brain barrier (BBB), processes that are implicated in the pathophysiology of AD and sleep fragmentation [13,17,18,19]. This convergence suggests that ozone may be an environmental modulator that, by affecting sleep architecture, could indirectly facilitate neurodegeneration. Climate change, by fostering air conditions that elevate ozone levels, may further intensify this exposure. World Health Organization estimations indicate that from 2030 to 2050, climate change-related diseases may result in up to 250,000 fatalities per year, impacting both cognitive health and restful sleep [13,15]. While certain meta-analyses have failed to establish a statistically significant correlation between ozone exposure and dementia risk, unlike other pollutants such as PM_2.5_ or NO_2_, these findings underscore the necessity for more targeted research on the specific impacts of ozone on the central nervous system (CNS) [20]. A tripartite relationship has been proposed in which ozone acts as an environmental exposure, AD manifests as a neurodegenerative condition, and sleep disorders function as a clinical and physiological connection. The three components converge on a shared axis defined by inflammation, synaptic dysfunction, and circadian disruption, emphasising ozone as a critical element for preventive and therapeutic efforts aimed at alleviating the decline associated with AD [21,22].

It is important to distinguish between the effects of ambient, uncontrolled tropospheric ozone exposure, the focus of this review, and the therapeutic use of medical ozone, which is administered at controlled doses via defined routes (e.g., ex vivo ozonation of blood or parenteral administration) and has been explored for its potential adaptive and antioxidant-priming properties. These are different exposure paradigms, and we do not assume that they share a single dose–response relationship; this distinction is developed in Section 2.6.

Literature from PubMed, Scopus and Web of Science from the year 1994 to 2025 was searched using combinations of the terms “tropospheric ozone,” “Alzheimer’s disease,” “sleep disorders,” “circadian rhythm,” “oxidative stress,” “neuroinflammation,” and “blood-brain barrier”. Studies were selected by the authors on the basis of relevance to the proposed mechanisms: we focused on primary experimental studies, epidemiological/population-based studies, and mechanistic reviews reporting measurable ozone exposure alongside neurological, cognitive, or sleep outcomes; studies addressing only stratospheric ozone or unrelated pollutants without a direct ozone component were excluded unless cited for comparative context. Background references on sleep, circadian biology and AD pathophysiology are cited for context and not as evidence of ozone effects. Because selection was based on expert judgment rather than a reproducible protocol, relevant studies may have been omitted, and this review should not be read as an exhaustive account of the ozone–sleep–AD literature. No formal risk-of-bias instrument was applied; methodological limitations of individual studies (e.g., exposure concentration, species, route of administration) are addressed in the text where relevant. Four of the 113 references (4.4%) correspond to prior work by members of the authors’ research group on ozone neurotoxicity [23,24,25,26,27]; they were included for thematic and methodological relevance, and readers should take this overlap into account when evaluating the selection.

The review is structured along the proposed pathway: oxidative and bioenergetic injury (Section 2.1), systemic and central inflammatory signaling (Section 2.2), neurotransmitter and sleep-architecture changes (Section 2.3), circadian disruption as a candidate effector (Section 2.4), supporting experimental and population evidence (Section 2.5), and the question of dose and hormesis (Section 2.6).

## 2. Ozone-Induced Mechanisms in Alzheimer’s Disease and Sleep Disruption

Ozone is a highly reactive oxidizing gas that, while it does not readily penetrate the BBB, can elicit systemic inflammatory responses with considerable effects on the CNS [17,28]. Prolonged exposure to inhaled pollutants, including vehicle emissions, has been associated with increased BBB permeability through activation of matrix metalloproteinases and loss of tight-junction proteins in mice [29], and BBB disruption has been reported in rats exposed to ozone combined with heat stress [17]. Such disruption could facilitate the entry of inflammatory mediators and peripheral immune cells, fostering a neuroinflammatory milieu that may support the onset and advancement of AD [30]. Ozone has also been reported to dysregulate hypothalamic neuroendocrine axes [31], and pollutant-related redox changes have been proposed to alter circadian regulation [32]; whether these effects reach the SCN has not been directly shown (Section 2.4). In the general sleep literature, circadian and sleep disruption are associated with Aβ and tau accumulation [33,34,35]. Particulate co-pollutants can additionally reach the CNS through the olfactory pathway or by crossing respiratory or gastrointestinal epithelial barriers [36]; this is relevant to co-exposure, whereas ozone itself, being a gas, is not expected to follow that route.

### 2.1. Reactive Oxygen Species and Bioenergetics

**Figure 1:**
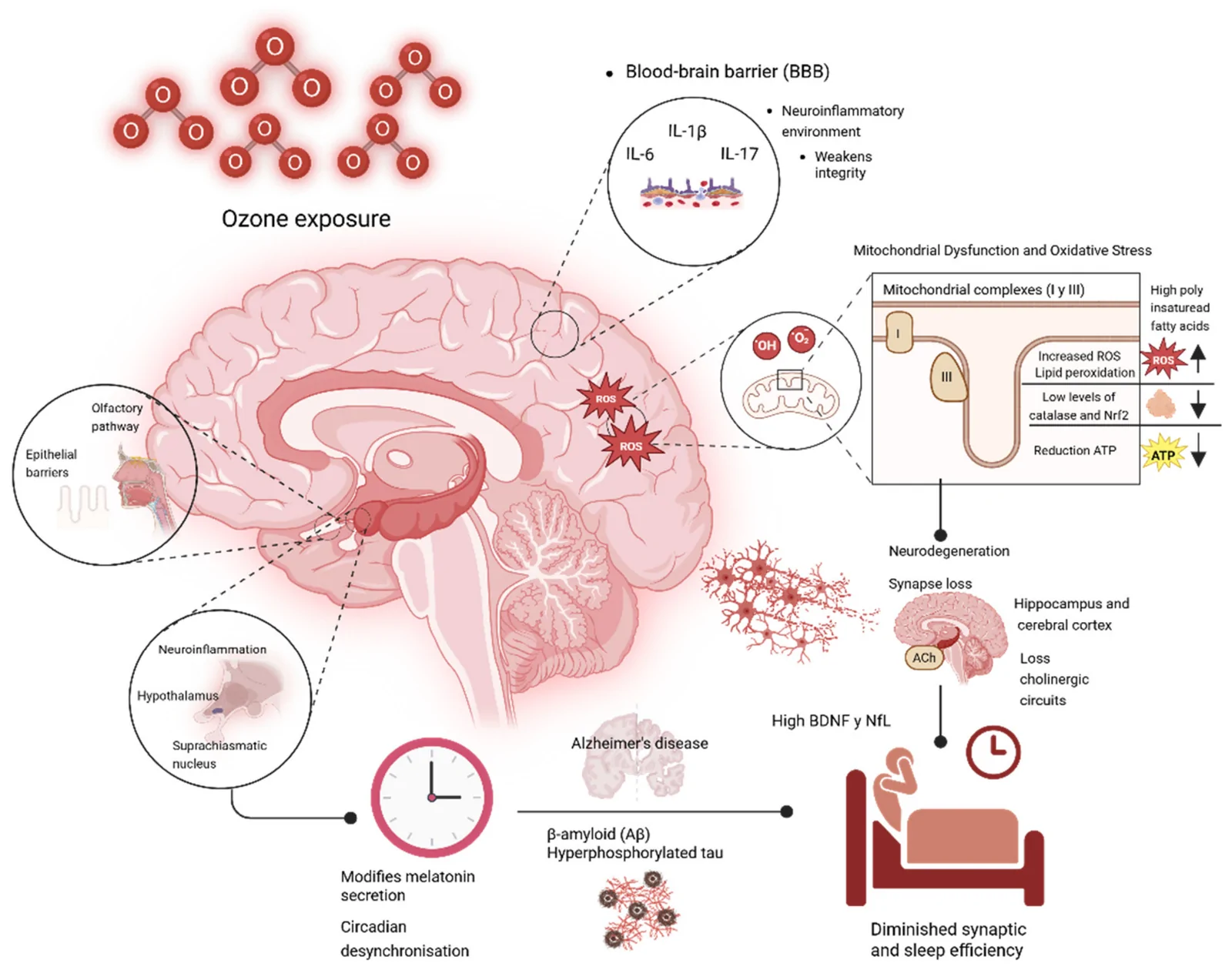
Proposed mechanisms by which ozone exposure contributes to neurodegeneration and sleep disruption in Alzheimer’s disease. Ozone exposure activates a neuroinflammatory response involving cytokines such as interleukins (IL-6, IL-1β, and IL-17), which may compromise the integrity of the blood–brain barrier and allow the entry of immune cells. Oxidative stress, increased ROS production and mitochondrial dysfunction, especially in complexes I and III, together with reduced antioxidant activity (catalase, Nrf2) are proposed to facilitate β-amyloid aggregation, tau hyperphosphorylation and synaptic loss. Effects on the hypothalamus/suprachiasmatic nucleus and on melatonin secretion are hypothesized and have not been directly shown for ozone. Altered BDNF and NfL levels and reduced acetylcholine are interpreted as markers of neuronal damage or compensatory responses. Created in BioRender. Garcia, C. (2026) https://BioRender.com/4eq256m.

Ozone exposure is also associated with impaired neuronal bioenergetics. Its interaction with the respiratory system produces reactive oxygen species (ROS), including hydrogen peroxide, hydroxyl radicals, and superoxide anions [37,38], which may penetrate the CNS via the blood flow [23] and trigger mitochondrial dysfunction [39]. Impairment of the electron transport chain, particularly of complexes I and III, has been reported in lung and liver after ozone exposure and has been proposed for the brain [25,40,41]. Increased ROS production and lipid peroxidation are expected to be particularly damaging in the brain, which is vulnerable because of its high content of polyunsaturated fatty acids and relatively limited antioxidant capacity; ozone exposure has been reported to reduce catalase activity and the nuclear factor erythroid 2-related factor 2 (Nrf2)-mediated antioxidant response [26,42]. These alterations are proposed to contribute to neurodegeneration and synapse loss, mechanisms relevant to AD pathophysiology [19,43]. Ozone has also been reported to promote the structural transformation of the Aβ1-40 peptide into insoluble forms [44]. The reduction in mitochondrial adenosine triphosphate (ATP) and the persistent elevation of ROS are proposed to impair the function of regions such as the hippocampus and cerebral cortex, which are critical for memory and sleep regulation [15,45,46]. Figure 1 depicts this integrated cascade, from ozone-induced BBB disruption through oxidative and mitochondrial damage to the resulting β-amyloid and tau pathology. 

Ozone-induced oxidative damage and mitochondrial dysfunction impair synaptic plasticity and diminish neurogenesis, impacting hippocampus and cholinergic circuits vital for memory and sleep [47]. Chronic ozone exposure in animal models results in cognitive deficits, diminished social interaction, and heightened immobility, indicative of structural changes including dendritic spine loss and disruption of brain networks associated with AD [48]. Acute ozone exposure in older people correlates with heightened levels of both neurofilament light chain (NfL), a marker of axonal damage, and brain-derived neurotrophic factor (BDNF), which may reflect either subclinical neuronal injury or a compensatory neuroplastic response, together indicating heightened susceptibility to cognitive and sleep problems [49]. In a mouse model of AD, sleep deprivation impairs memory, tau metabolism and synaptic integrity [50], illustrating how sleep loss could amplify neuronal vulnerability; this evidence is not ozone-specific.

Because oxidative injury in peripheral tissues is accompanied by a systemic inflammatory response, the next section examines the inflammatory signal that is proposed to link peripheral exposure to central effects.

### 2.2. Systemic and Central Inflammatory, Neuroendocrine, and Gut–Brain Mechanisms

In experimental models, ozone stimulates glial activation, with an increase in microglia and astrocytes, which initiates chronic inflammatory cascades via cytokines such as interleukins (IL) -1β, -17, and -6, accompanied by a reduction in IL-10 [51]. Neuroinflammation is proposed to promote Aβ accumulation and tau phosphorylation and to interfere with the processes that govern non-REM sleep; conversely, sleep loss and sleep restriction increase this inflammatory response alongside Aβ accumulation in rodents [52,53], suggesting a potentially self-reinforcing cycle (these sleep studies are not ozone-specific). In residents of severely polluted regions, such as Mexico City, cyclooxygenase-2 (COX-2) overexpression and amyloid-beta 42 (Aβ42) accumulation have been described in young individuals with no symptoms [54,55]; because these populations are exposed to complex pollutant mixtures, the specific contribution of ozone cannot be isolated, but the findings are compatible with air pollution-related inflammation initiating neurodegenerative processes from early stages.

This glial response may be compounded by systemic neuroendocrine dysregulation. Ozone exposure activates the hypothalamic–pituitary–adrenal and sympathetic–adrenal–medullary axis, resulting in the release of glucocorticoids and catecholamines that modify the neuroendocrine and immunological milieu; in rats, this effect requires adrenal-derived stress hormones [31,56], and similar neuroendocrine and inflammatory changes have been reported with traffic-related particulate matter [55]. High-mobility group B1 (HMGB1) has been proposed as a mediator that engages microglial receptors such as triggering receptors expressed on myeloid cells 2 (TREM2). TREM2 impairment is associated with diminished immunological responsiveness and heightened Aβ pathology and ozone has been shown to dysregulate the peri-plaque microenvironment in an amyloid pathology model [57,58,59]. The macrophage-1 antigen/nicotinamide adenine dinucleotide phosphate oxidase 2 (MAC1/NOX2) and the extracellular signal-regulated kinase 1 and 2 (ERK1/2) pathway, which sustains chronic microglial inflammation in other models, is considered a likely mechanism of ozone-related neuroinflammation, though direct evidence in ozone models is limited [60,61,62]. Additionally, lipid peroxidation by-products, including malondialdehyde (MDA) and 4-hydroxynonenal (HNE), are thought to cross into the brain and further exacerbate neuroinflammation [63,64,65].

**Figure 2:**
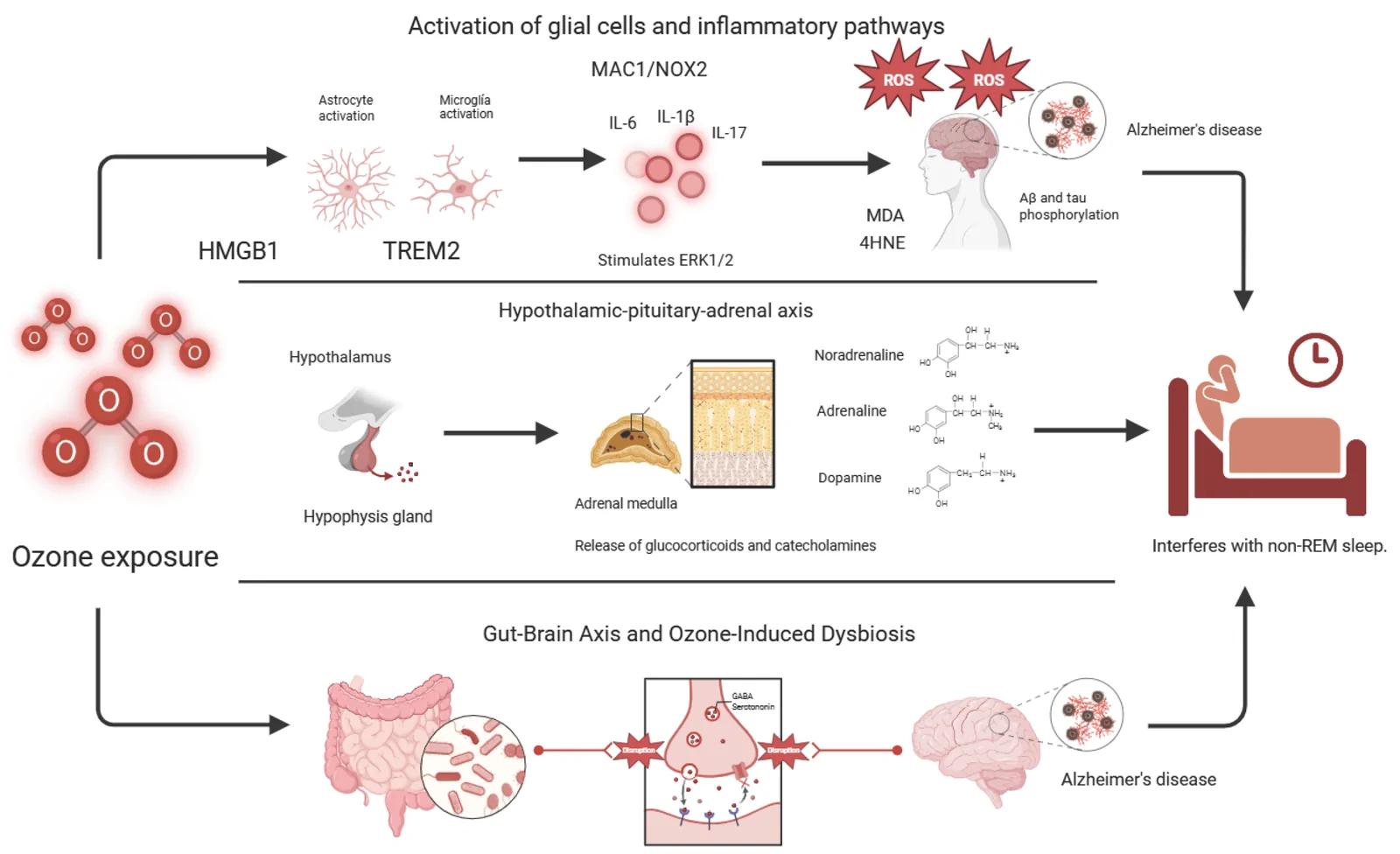
Proposed inflammatory, neuroendocrine and gut–brain mechanisms linking ozone exposure to Alzheimer’s disease and sleep disturbances. Ozone exposure is associated with glial activation (astrocytes and microglia) and proinflammatory cytokines; the MAC1/NOX2 pathway, is hypothesized to increase ROS, MDA, and 4HNE, which may favor tau phosphorylation and Aβ accumulation. Ozone also stimulates the hypothalamic–pituitary–adrenal axis with release of glucocorticoids and catecholamines, and HMGB1 and TREM2 have been proposed as mediators of the microglial response. Likewise, changes in gut microbiota may impact neurotransmitters (GABA, 5-HT) through the gut–brain axis. These interconnected processes are hypothesized to promote neurodegeneration and non-REM sleep disturbances. Created by BioRender. Garcia, C. (2026) https://BioRender.com/4eq256m.

This inflammatory and neuroendocrine burden may extend beyond the brain itself: ozone has been associated with changes in the gut microbiota, leading to dysbiosis that induces systemic and cerebral inflammation via the gut–brain axis [66]. In addition to the inflammatory process, the increase in 7α-hydroxy-3-oxo-4-cholestenoate (also known as 7-HOCA) following ozone exposure may be linked to such alterations in the gut microbiota, given that gut microorganisms are involved in the production of secondary bile acids and 7-HOCA is an intermediate in this process [67]. Dysbiosis is also associated with alterations in neurotransmitters such gamma-aminobutyric acid (GABA) and serotonin (5-HT), which are important for regulating sleep and cognitive function, both of which are significantly disrupted in AD [68,69]. It has been reported that GABA is synthesized by certain gut bacteria, which can utilize it for their own benefit [70]. It has also been suggested that these bacteria can act on dietary glutamate and exert beneficial health effects [71]. Altogether, these findings are compatible with ozone contributing to inflammatory, neuroendocrine and gut–brain alterations that may be relevant to AD and sleep disorders, although much of the mechanistic support derives from indirect or non-ozone models. These inflammatory and neuroendocrine changes are proposed to feed into the neurochemical systems that govern sleep, which are considered next. Figure 2 illustrates these proposed pathways.

### 2.3. Neurotransmitter Systems and Sleep Architecture

The inflammatory and neuroendocrine changes described above are proposed to feed into the neurochemical systems that govern sleep. Sleep fragmentation and circadian disruption are common in AD and are associated with Aβ pathology; these studies are not ozone-specific, and whether ozone intensifies these disturbances in people with AD has not, to our knowledge, been directly tested [72,73,74].

In rodents, ozone exposure is associated with region-specific changes in serotonin and its metabolite [75] and with increased serotonin content in the pons [76], and with reduced acetylcholine levels in the medial preoptic area, accompanied by a decrease in paradoxical (REM) sleep [27]. Ozone has also been reported to increase the plasma kynurenine-to-tryptophan (KYN/TRP) ratio and to alter hippocampal serotonin receptor and neurotrophic factor expression, with a role for glucocorticoid-mediated mechanisms [77]. Evidence that ozone alters dopamine, norepinephrine or GABA in sleep-relevant circuits is, in the sources we cite, indirect (for example, GABA and serotonin changes proposed through the gut–brain axis (Section 2.2).

Independently of ozone, fragmented sleep reduces glymphatic function, which can impair the clearance of Aβ and tau and exacerbate neuroinflammation [78,79,80,81]. Pollutant exposure has been proposed to compromise glymphatic function [36,82,83], but we are not aware of studies testing this directly for ozone. A bidirectional relationship is therefore plausible, in which ozone-related inflammation alters sleep and altered sleep amplifies neuroinflammatory and amyloid pathology, but the available data do not demonstrate this loop for ozone.

Circadian regulation, the other axis along which ozone could affect sleep, is considered next.

### 2.4. Circadian Disruption as a Candidate Effector

Evidence that ozone perturbs the circadian machinery comes mainly from peripheral tissue. In mouse lung, ozone altered expression of the clock genes Bmal1, Per1–3, Cry2, Fbxl3, Npas2, Nr1d1, Btrc and Prkaa1, with the Notch pathway (Notch3/4) proposed as an upstream regulator [84]; acute and repeated ozone exposures also differentially affected clock-gene expression in mice [85]. Reviews propose that pollution-related redox changes can alter circadian regulation and that clock genes are implicated in inflammation and tissue repair [32,86].

For the CNS, the evidence is indirect. Ozone has been reported to dysregulate hypothalamic neuroendocrine axes through adrenal-derived stress hormones [31], and it alters sleep–wake architecture in rodents [75,76,87]. However, changes in sleep architecture are not equivalent to circadian disruption, and in the studies we cite we did not identify direct measurements of SCN clock-gene expression, SCN activity, or circadian outputs (e.g., rhythms of locomotor activity, body temperature or melatonin) after ozone exposure. The role of the SCN as master pacemaker [8,88,89] and the neuroprotective actions of melatonin [44,90,91,92] are background physiology and should not be read as evidence of ozone-induced SCN damage or melatonin reduction.

Establishing circadian disruption as a mediator of the ozone–AD relationship, researchers must first demonstrate temporal precedence, showing that rhythm or clock disruption—measured via actigraphy, melatonin onset, or SCN-specific expression—precedes AD-relevant changes such as Aβ accumulation, p-tau, and impaired glymphatic clearance [93,94]. This must be accompanied by a graded exposure–response relationship across both the mediator and the outcome, alongside formal causal mediation analysis in longitudinal cohorts with repeated measures that strictly control for co-pollutants and exposure–mediator confounding. Finally, experimental manipulation must confirm that preventing circadian disruption through light entrainment, melatonin replacement, or clock-gene manipulation attenuates downstream pathology under equal ozone exposure, whereas inducing disruption without ozone reproduces it. To our knowledge, no study has yet deployed these rigorous designs for ozone, leaving current data unable to distinguish true mediation from mere co-occurrence in a relationship that is also likely bidirectional, given that AD pathology inherently disrupts sleep and circadian rhythms [5,48,50].

**Figure 3:**
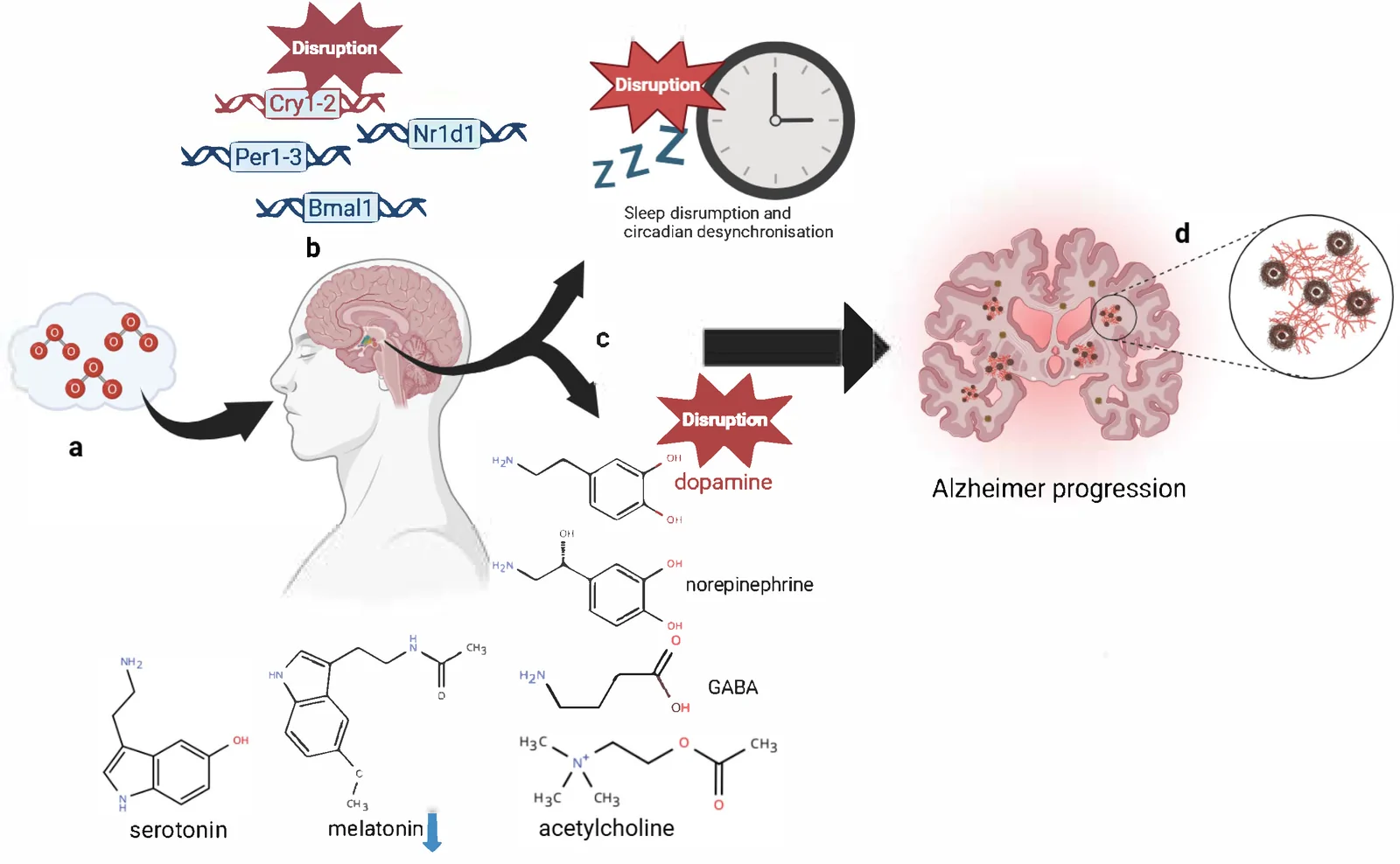
Proposed circadian and neurochemical effects of ozone. Ozone exposure (**a**) alters clock-gene expression (Bmal1, Per1–3, Cry1–2, Nr1d1), as shown in lung [50,52] and hypothesized for the SCN; (**b**) may alter the secretion and activity of neurotransmitters involved in sleep regulation; which could (**c**) sustain a neuroinflammatory response and (**d**) promote Aβ and tau accumulation. Panels (**c**,**d**) represent hypothesized links. Created by BioRender. Garcia, C. (2026) https://BioRender.com/4eq256m.

We therefore propose that circadian disturbance could contribute to the effects of ozone by loosening rhythmic control of innate immune signaling and by impairing sleep-dependent clearance of Aβ and tau [78,79]. This inference derives from general chronobiology and sleep research and has not been demonstrated for ozone. Circadian disruption remains one of several candidate effectors, alongside direct oxidative injury, neuroinflammation and neurotransmitter changes (Figure 3).

In summary, the proposed pathway (oxidative and bioenergetic injury, systemic and central inflammatory signaling, neurotransmitter changes, and a candidate circadian component) is supported at its individual nodes mainly by experimental studies in rodents at high concentrations and, to a lesser extent, by population studies; the links between nodes remain largely hypothetical.

### 2.5. Experimental and Population-Based Evidence

Experimental and population studies have reported associations of variable strength between air pollutant exposure, including tropospheric ozone, sleep disturbances, and early neuropathological alterations related to AD; primary studies with quantified ozone exposure are summarized in Table 1. In severely polluted metropolitan populations, research indicates that children and adolescents chronically exposed to complex pollutant mixtures that include ozone and ultrafine particles show disruptions in the sleep–wake cycle and damage to brainstem nuclei involved in REM sleep. These modifications influence sleep architecture and are correlated with the early emergence of neuropathological biomarkers indicative of AD, including Aβ and hyperphosphorylated tau deposits [95,96].

A high prevalence of sleep disorders, such as insomnia, restless legs syndrome, and REM sleep behaviour disorder, has been documented in young individuals exposed to elevated air pollution levels. These symptoms have been observed alongside initial neuropathological indicators linked to neurodegenerative disorders, indicating a direct impact of pollutants on the brain circuits governing sleep and cognitive functions [82,96], but they do not establish that sleep disturbance caused by environmental exposure is a causal risk factor for AD.

A population study in China involving 395,651 older adults found that short-term exposure to ozone and sulfur dioxide was associated with more hospital visits for sleep disorders, with the strongest ozone association at a lag of 5 days [97]. These data indicate a short-term association with sleep-related health outcomes; they do not by themselves show that ozone promotes neurodegenerative processes.

At a broader ecological level, global ozonesonde and surface observations show tropospheric ozone increasing by an average of 1.8 ppb per decade since the 1990s [98], and GBD 2021 estimates report a rise in age-standardized AD and dementia prevalence and incidence over the same period [3]. These parallel trends are ecological, subject to many confounders, and do not establish causality; they highlight the need for integrative research on how chronic exposure to air pollutants may contribute to neurodegenerative risk.

**Table 1:** Key experimental and population-based evidence on ozone exposure, neurological outcomes, and sleep.

Study/Reference	Design	Population/Model	Verified O_3_ Dose/Concentration	Main Finding
**Experimental (animal)**				
Huitrón-Reséndiz et al. 1994 [75]	Experimental, rat	Male Wistar n = 22	1.5 ppm, 2 h/day, monitoring during subsequent 22 h	Regional serotonin and metabolite changes after ozone exposure
Paz and Huitrón-Reséndiz 1996 [76]	Experimental, rat	Polysomnographic recording	0.35/0.75/1.50 ppm	↓ REM at 2 h, ↑ slow-wave sleep at 12 h, dose-dependent ↑ pontine serotonin
Alfaro-Rodríguez and González-Piña 2005 [87]	Experimental, rat	Male Wistar n = 10	0.5 ppm, 24 h of a single continuous exposure	Paradoxical (REM) sleep decrease related to reduced acetylcholine in the medial preoptic area
Velázquez-Pérez et al. 2021 [51]	Experimental, rat	Male Wistar, n = 6/group	0.25 ppm, 4 h/day, 7–90 days	P2X7 receptor changes, hippocampal neuroinflammation and neurodegeneration
Rose et al. 2020 [77]	Experimental, rat	Male Fischer n = 5/group	0.8 ppm, 4 h of a single acute exposure	↑ plasma kynurenine/tryptophan ratio; altered hippocampal serotonin receptor and neurotrophic factor expression; role of stress hormones
Henríquez et al. 2019 [31]	Experimental, rat	Male Wistar-Kyoto (WKY) n = 6–8/group for serum/physiology; n = 4/group for transcriptomics	0.8 ppm, 4 h of a single acute exposure	Neuroendocrine axis dysregulation requiring adrenal-derived stress hormones
Greve et al. 2023 [59]	Experimental in vivo, mice	Male Transgenic 5xFAD and wild-type (WT) n = 5–12/group for exposure cohorts; n = 5/group for proteomics; n = 5–8/group for mechanistic/rescue experiments	0.3 and 1.0 ppm, 4 h per day/4 weeks, subchronic exposure	Ozone dysregulates the peri-plaque microenvironment
Yang et al. 2025 [18]	Experimental, mouse	Male C57BL/6 n = 3–4/group, experimental; n = 10–12, behavioral; n = 3–4, molecular analysis	0.6 ppm, 4 h/day, 30 days	Neurodegenerative alterations with mitochondrial dysfunction in glial cells
Dai et al. 2025 [19]	Experimental, mouse	Male C57BL/6 n = 6–10/group, conduct and molecular analysis	1.0 ppm, 3 h/day, 28 days	Simultaneous co-exposure to ozone (O_3_) and polystyrene nanoplastics (PS-NPs) that produces a synergistic and aggravated neurotoxic effect
Zhang et al. 2025 [84]	Experimental, mouse lung; in silico	Male C57BL/6 and WT n = 12 (6/group: control and ozone exposure)	1.0 ppm, 3 h of a single continuous exposure	Ozone inhalation disrupts the lung’s biological clock by abnormally activating the Notch signaling pathway
Sundar et al. 2023 [85]	Experimental, mouse lung	Male and female C57BL/6 n = 4–6/group: acute and subacute	2.0 ppm, 3 h of a single acute exposure; 0.8 ppm, subacute exposure, 9 days	Ozone exposure causes a drastic and “sex-dependent” disruption of circadian clock genes, with radically opposite effects in alveolar macrophages
**Experimental (therapeutic ozone; not evidence of ambient toxicity)**				
Yan YN et al. 2022 [99]	Experimental, rat	REM sleep deprivation model	1.1 mg/kg, IP, 28 days (O_2_–O_3_ mix, 30 µg/ml)	Ozone reduced spatial memory deficits and hippocampal Sema3A expression
**Population-based (human)**				
Qu R et al. 2023 [49]	Panel study	Healthy elderly, Xinxiang China	Ambient; +10 µg/m^3^ → +74% BDNF, +197% NfL	Acute ozone exposure and serum neural damage biomarkers
Tang et al. 2020 [97]	Population time-series	n = 395,651 elderly, Ningbo, China	Ambient; daily maximum 8-h average concentration of O_3_, evaluating the relative risk of hospital visits per every 10 μg/m^3^ increase in ozone levels.	O_3_/SO_2_ association with hospital visits for sleep disorders, strongest at lag 5 days
Cleary et al. 2018 [100]	Longitudinal cohort study utilizing a spatiotemporal Bayesian hierarchical model	n = 5116 male and female older adults, from the National Alzheimer’s Coordinating Center (NACC) possessing normal baseline cognition, later transitioned toward cognitive decline, USA	Long-term residential ambient air quality by specifically tracking chronic exposure to low-level ozone (averaging ~20–25 ppb) from 2004 to 2008	Ozone, but not PM_2.5_, associated with accelerated cognitive decline; stronger in APOE4 carriers
Zhang et al. 2023 [12]	Mixed design combining two analytical approaches: DID framework and micro-level longitudinal observational study	n = 90 participants (male and female young adults included) followed continuously drawn from the China Family Panel Studies (CFPS) database	Ozone concentration measured using (IQR increments derived from real-world ambient background levels, tracked by three time scales in parallel: yearly average, monthly average and weekly/daily windows	Ground-level ozone causally reduces sleep time and alters brain resting-state activity via a quasi-linear dose–response curve with no safe lower threshold.

Primary studies with quantified ozone exposure that are cited in Section 2.5 and elsewhere in this review as direct evidence of ozone effects on neurological, cognitive, sleep/circadian or neuroendocrine outcomes, ordered by study design. Reviews, background references and studies of mixed pollutants without an ozone-specific analysis are not listed, and the table is not an exhaustive compilation. For experimental studies, ozone concentrations and exposure regimens are reported as stated in the original publications; for population studies, ambient exposure metrics and effect estimates are reported where available. Arrows denote the direction of change reported relative to control or baseline conditions: ↑, increase; ↓, decrease. Abbreviations: BBB, blood–brain barrier; BDNF, brain-derived neurotrophic factor; h, hour(s); IP, intraperitoneal; mg/kg, milligrams per kilogram body weight; n, sample size; NfL, neurofilament light chain; O_2_–O_3_, oxygen–ozone mixture; O_3_, ozone; P2X7, purinergic receptor P2X7; ppm, parts per million; REM, rapid eye movement sleep; Sema3A, semaphorin 3A; SO_2_, sulfur dioxide; µg/m^3^, micrograms per cubic meter.

Findings in animal models are consistent with an effect of ozone on sleep. In rats exposed to ozone at 0.35–1.50 ppm, REM sleep decreased early (at 2 h) and slow-wave sleep increased later (at 12 h) in a dose-dependent manner, accompanied by increased serotonin in the pons, a region involved in sleep–wake regulation [76]. This was suggested to reflect pontine dysfunction mediated by proinflammatory compounds such as prostaglandins [76]. Similar sleep alterations with regional changes in serotonin and its metabolite [75], and a reduction in paradoxical sleep associated with lower acetylcholine in the medial preoptic area [87], have been reported in rats.

Population studies of air pollutants, including ozone, and cognitive and sleep-related outcomes report associations that are heterogeneous across pollutants, cohorts and outcomes [101,102]. Independently of ozone, reduced slow-wave activity in NREM sleep, as in obstructive sleep apnea with intermittent hypoxia, is associated with higher Aβ42 and total tau, including in people without prior cognitive impairment [103,104,105]. This supports a link between sleep disturbance and AD biomarkers in general, which is the premise on which a sleep-mediated ozone effect would rest, but the link has not been tested specifically for ozone.

Collectively, experimental and population evidence suggests that tropospheric ozone acts as an environmental modulator that negatively impacts sleep quality, which in turn could contribute to neuropathological alterations characteristic of AD, but these links have not been demonstrated for ozone in humans.

### 2.6. Dose, Magnitude of Exposure, and Hormesis

A critical variable in interpreting this literature is the magnitude of the ozone concentrations employed in experimental models relative to real-world ambient exposure. Rodent inhalation studies cited here used concentrations of roughly 0.25–1.5 ppm [17,76], approximately 3.6- to 21-fold the regulatory ambient standard of 0.070 ppm (8-h average) set by the US EPA [106]. Experimental exposures are also usually acute or subchronic and do not replicate the chronic low-dose exposure of human populations. Direct experimental evidence on dose–response across a physiologically realistic range is scarce, and the available studies were not designed to resolve it. Extrapolation from high-dose experimental findings to chronic low-level ambient exposure should therefore be made with caution; we treat this as a central caveat of the review rather than a peripheral note [51].

Three concepts are distinguished here: first, the dose dependence of ozone toxicity; second, hormesis, which we use strictly for a biphasic dose–response in which low exposures elicit adaptive responses (e.g., Nrf2 activation) and higher exposures cause injury; and third, therapeutic medical ozone, which is not low-dose inhalation: clinical applications rely on ex vivo ozonation of autologous blood followed by reinfusion, a controlled and transient oxidative eustress [107,108], and experimental protocols use parenteral administration, such as intraperitoneal ozone in a rat model of REM sleep deprivation, in which ozone reduced memory deficits and hippocampal Sema3A expression [99]. These are different exposure paradigms with different routes, tissue doses and reaction substrates, and we do not treat them as a single phenomenon. For this reason, ex vivo and parenteral ozonation data are not used here to infer the dose–response of inhaled ambient ozone. The studies on therapeutic ozone that we cite [109,110,111] used single-dose protocols and cannot establish a hormetic curve.

Whether low ambient ozone exposure is inert, adaptive or harmful is untested, and a protective low-dose effect is a hypothesis, not a finding. The one low-level ambient exposure study we cite, in older adults, reported an association with faster cognitive decline [100], which argues against assuming that ambient exposures are benign. Direct data on low-dose inhaled ozone and neurological outcomes in humans are lacking, and distinguishing the pathogenic effects of ambient ozone from the effects of controlled medical ozone requires dose-graded studies.

## 3. Discussion

This review synthesizes experimental and observational evidence that is compatible with the hypothesis that tropospheric ozone exposure contributes to AD-related neurodegeneration and sleep disruption through a systemic oxidative–inflammatory pathway. In experimental models, ozone exposure is associated with oxidative stress, mitochondrial dysfunction, glial activation and cytokine production [18], BBB alterations [17], and changes in serotonergic and cholinergic systems [60,61,62,112], with effects on sleep architecture in rodents [75,76,87]. These experimental findings come mainly from rodents exposed to concentrations above regulatory ambient standards, and in some studies ozone was combined with other exposures [17,18].

Several links in the proposed pathway are not established. Clock-gene changes have been shown mainly in peripheral tissue [84,85]; direct evidence of ozone effects on the SCN or on circadian outputs is limited, and the data cannot distinguish circadian disruption as a mediator from co-occurrence (Section 2.4). Sleep and neurodegeneration are likely to influence each other [48], so a unidirectional model is probably an oversimplification. Moreover, support for several steps (MAC1/NOX2, HMGB1/TREM2, lipid peroxidation by-products, gut–brain signaling) comes from non-ozone or indirect models (Section 2.2).

Epidemiological studies have reported associations, though not uniformly across cohorts, between elevated ozone levels and increased prevalence of sleep disorders or cognitive decline, particularly in vulnerable populations such as older adults [97,100,102]. In one cohort of older adults, ozone but not PM2.5 was associated with faster cognitive decline, with a stronger association in APOE4 carriers [100], suggesting possible pollutant specificity and gene–environment interaction. At the same time, meta-analytic evidence for ozone and dementia is inconsistent compared with PM_2.5_ or NO_2_ [20]. This discrepancy between a plausible mechanism and an inconsistent epidemiological signal may reflect several non-mutually exclusive factors: (i) exposure misclassification, since ambient ozone monitoring stations rarely capture individual indoor/outdoor exposure; (ii) confounding by co-pollutants, given that ozone rarely occurs in isolation from PM_2.5_ and NO_2_ in urban settings; (iii) differences in latency windows, as neurodegenerative outcomes may require decades of cumulative exposure while most cohorts have shorter follow-up; (iv) outcome heterogeneity, with some studies assessing clinical dementia and others subclinical biomarkers or sleep-specific endpoints; and (v) the possibility that the effect of ambient ozone at low concentrations is weaker than that inferred from high-dose experiments. We cannot determine from the available data which of these explains the gap, and methodological limitations alone should not be assumed to account for it.

The relation between ambient ozone and medically administered ozone is discussed in Section 2.6. We emphasize that these are different exposure paradigms, that controlled medical ozone does not provide evidence about the dose–response of inhaled ozone, and that the hormetic interpretation of the experimental-to-ambient gap remains a hypothesis. Several limitations should be acknowledged, including heterogeneity in exposure assessment, confounding by co-pollutants, and reliance on ecological or observational data in human studies. Most of the experimental evidence summarized here derives from rodent models exposed to roughly 0.25–1.5 ppm, which is substantially above the regulatory ambient standard (0.070 ppm, 8-h average) [51]. These studies often utilize acute or subchronic protocols that do not replicate chronic, low-dose human exposure, thereby limiting the direct extrapolation of mechanistic findings to real-world scenarios. The reviewed literature also rarely disaggregates findings by sex, despite evidence that neuroinflammatory and glymphatic responses to environmental insults can differ between males and females; consequently, future studies should examine sex as a biological variable. As a narrative review, this work does not include a formal quality assessment nor did it follow a predefined, recorded search protocol. The selection of studies was based on the authors’ judgment and is not exhaustive or reproducible; thus, relevant studies—including earlier ozone–sleep work in rodents—may have been omitted, which could bias the synthesis toward the mechanisms and models discussed here.

Future research should prioritize longitudinal studies with individualized exposure monitoring, objective sleep and circadian measurements, and biomarker profiling. Furthermore, researchers should adopt exposure-graded designs that allow for the examination of dose–response relationships and temporal sequences, including formal mediation analysis. Experimental studies utilizing ozone concentrations closer to ambient exposure, as well as direct measurements of SCN function and circadian outputs after exposure, are critically needed. Interventions to improve air quality and sleep health are reasonable public-health goals in their own right [48,112,113]; however, whether they would reduce AD risk through the ozone pathway remains to be established.

## 4. Conclusions

In experimental models, ozone exposure is associated with oxidative stress, neuroinflammation, mitochondrial dysfunction, neurotransmitter changes and altered sleep, and with alterations relevant to AD pathology. We propose, as a working hypothesis, that these effects arise from a systemic oxidative–inflammatory signal that reaches the central nervous system, with sleep/circadian disruption and AD-related pathology as downstream outcomes that may reinforce each other. Circadian disruption is a candidate effector, not an established mediator, and current data cannot distinguish mediation from co-occurrence. Given the substantial gap between the ozone concentrations used in experimental models and real-world ambient exposure, together with the inconsistent epidemiological signal for ozone relative to other pollutants such as PM_2.5_ and NO_2_, ambient ozone should at present be regarded as a biologically plausible, but not yet epidemiologically confirmed, candidate risk factor for AD-related neurodegeneration and sleep disturbance. Exposure-graded longitudinal studies, with formal mediation analysis, are an essential next step before reducing ozone exposure and safeguarding sleep can be confidently incorporated into air quality policy and clinical screening aimed at preserving cognitive function.

## Data Availability

No new data were created or analyzed in this study. Data sharing is not applicable to this article.

## Abbreviations

The following abbreviations are used in this manuscript:

Aβ/Aβ42	Amyloid-beta/amyloid-beta 42
AD	Alzheimer’s disease
APOE4	Apolipoprotein E ε4
ATP	Adenosine triphosphate
BBB	Blood–brain barrier
BDNF	Brain-derived neurotrophic factor
CNS	Central nervous system
COX-2	Cyclooxygenase-2
ERK1/2	Extracellular signal-regulated kinase 1/2
GABA	Gamma-aminobutyric acid
GBD	Global Burden of Disease
h	Hour(s) (Table 1)
5-HT	5-hydroxytryptamine (serotonin)
HMGB1	High-mobility group box 1
HPA	Hypothalamic–pituitary–adrenal (axis)
IL-1β, IL-6, IL-10, IL-17	Interleukin-1β, -6, -10, -17
IP	Intraperitoneal
KYN/TRP	Kynurenine/tryptophan (ratio)
MAC1	Macrophage antigen complex-1
mg/kg	Milligrams per kilogram
n	Sample size
NfL	Neurofilament light chain
NO_2_	Nitrogen dioxide
NOx	Nitrogen oxides
NOX2	NADPH oxidase 2
NREM	Non-rapid eye movement (sleep)
O_2_-O_3_	Oxygen-ozone (mixture)
O_3_	Ozone
P2X7	Purinergic receptor P2X7
PM_2.5_	Fine particulate matter (≤2.5 µm)
ppm	Parts per million
REM	Rapid eye movement (sleep)
ROS	Reactive oxygen species
SAM	Sympathetic–adrenal–medullary (axis)
SCN	Suprachiasmatic nucleus
Sema3A	Semaphorin 3A
SO_2_	Sulfur dioxide
T-Tau	Total tau
TREM2	Triggering receptor expressed on myeloid cells 2
µg/m^3^	Micrograms per cubic meter

## References

[1] Selkoe D.J., Hardy J. (2016). The amyloid hypothesis of Alzheimer’s disease at 25 years. EMBO Mol. Med..

[2] Samanta S., Bagchi D., Chaurasia R.N., Ohia S.E. (2024). Neurodegenerative diseases and circadian rhythm dysfunction: A bidirectional nexus. A Review on Diverse Neurological Disorders.

[3] Yu D.T., Li R.X., Sun J.R., Rong X.W., Guo X.G., Zhu G.D. (2025). Global mortality, prevalence and disability-adjusted life years of Alzheimer’s disease and other dementias in adults aged 60 years or older, and the impact of the COVID-19 pandemic: A comprehensive analysis for the global burden of disease 2021. BMC Psychiatry.

[4] Zhang X.X., Tian Y., Wang Z.T., Ma Y.H., Tan L., Yu J.T. (2021). The Epidemiology of Alzheimer’s Disease Modifiable Risk Factors and Prevention. J. Prev. Alzheimer’s Dis..

[5] Ju Y.E., Lucey B.P., Holtzman D.M. (2014). Sleep and Alzheimer disease pathology—A bidirectional relationship. Nat. Rev. Neurol..

[6] Choe Y.M., Byun M.S., Yi D., Lee J.H., Jeon S.Y., Sohn B.K., Kim Y.K., Shin S.A., Sohn C.H., Lee Y.J. (2019). Sleep experiences during different lifetime periods and in vivo Alzheimer pathologies. Alzheimer’s Res. Ther..

[7] Eckhardt J.L., Isenberg L., Aslanyan V., Monreal T., Stradford J., Fenton L., Contreras J.A., Mack W.J., Pa J. (2025). Circadian rhythms are associated with higher amyloid-β and tau and poorer cognition in older adults. Brain Commun..

[8] Moore R.Y. (2007). Suprachiasmatic nucleus in sleep-wake regulation. Sleep Med..

[9] Chokroverty S. (2010). Overview of sleep & sleep disorders. Indian J. Med. Res..

[10] Govindugari V.L., Golla S., Reddy S.D.M., Chunduri A., Nunna L.S.V., Madasu J., Shamshabad V., Bandela M., Suryadevara V. (2023). Thwarting Alzheimer’s disease through healthy lifestyle habits: Hope for the future. Neurol. Int..

[11] Younan D., Petkus A.J., Widaman K.F., Wang X., Casanova R., Espeland M.A., Gatz M., Henderson V.W., Manson J.E., Rapp S.R. (2020). Particulate matter and episodic memory decline mediated by early neuroanatomic biomarkers of Alzheimer’s disease. Brain.

[12] Zhang Y., Ke L., Ma X., Di Q. (2023). Impact of ground-level ozone exposure on sleep quality and electroencephalogram patterns at different time scales. Environ. Res..

[13] Aderinto N., Babalola A., Ajagbe A.O., Obasanjo O.M., Moradeyo A., Ukoaka B.M., Oyetola E.O., Babalola D.O., Abraham I.C., Olatunji G. (2025). Molecular basis of complex relationships between climate change, sleep disorders, and Alzheimer’s disease. Egypt. J. Neurol. Psychiatr. Neurosurg..

[14] Croze M.L., Zimmer L. (2018). Ozone atmospheric pollution and Alzheimer’s disease: From epidemiological facts to molecular mechanisms. J. Alzheimer’s Dis..

[15] Zhang F., Zhong R., Li S., Fu Z., Wang R., Wang T., Huang Z., Le W. (2019). Alteration in sleep architecture and electroencephalogram as an early sign of Alzheimer’s disease preceding the disease pathology and cognitive decline. Alzheimer’s Dement..

[16] Sîrbu C.A., Stefan I., Dumitru R., Mitrica M., Manole A.M., Vasile T.M., Stefani C., Ranetti A.E. (2022). Air pollution and its devastating effects on the central nervous system. Healthcare.

[17] Yan Z., Liu Y.M., Wu W.D., Jiang Y., Zhuo L.B. (2023). Combined exposure of heat stress and ozone enhanced cognitive impairment via neuroinflammation and blood brain barrier disruption in male rats. Sci. Total Environ..

[18] Yang L., Zhao S., Wu Q., Zeng Y., Zhang A., Sun H., Xu F., Shi S., Li J., An Z. (2025). Ozone and PM2.5 co-exposure induced neurodegenerative alterations in mice: Implication of mitochondrial dysfunction in glial cells. Environ. Int..

[19] Dai A., Liu X., Chen Y., Wang Y., Qi H., Zeng Y., Li J. (2025). Co-exposure to ozone and polystyrene nanoplastic exacerbates cognitive impairment and anxiety-like behavior by regulating neuronal pyroptosis in mice. Environ. Int..

[20] Jones A., Ali M.U., Mayhew A., Aryal K., Correia R.H., Dash D., Manis D.R., Rehman A., O’Connell M.E., Taler V. (2025). Environmental risk factors for all-cause dementia, Alzheimer’s disease dementia, vascular dementia, and mild cognitive impairment: An umbrella review and meta-analysis. Environ. Res..

[21] Singh S.A., Suresh S., Singh A., Chandran L., Vellapandian C. (2022). Perspectives of ozone induced neuropathology and memory decline in Alzheimer’s disease: A systematic review of preclinical evidences. Environ. Pollut..

[22] Lane M., Oyster E., Luo Y., Wang H. (2025). The effects of air pollution on neurological diseases: A narrative review on causes and mechanisms. Toxics.

[23] Martínez-Lazcano J.C., González-Guevara E., del Carmen Rubio M., Franco-Pérez J., Custodio V., Hernández-Cerón M., Livera C., Paz C. (2013). The effects of ozone exposure and associated injury mechanisms on the central nervous system. Rev. Neurosci..

[24] González-Guevara E., Martínez-Lazcano J.C., Custodio V., Hernández-Cerón M., Rubio C., Paz C. (2014). Exposure to ozone induces a systemic inflammatory response: Possible source of the neurological alterations induced by this gas. Inhal. Toxicol..

[25] Marin-Castañeda L.A., Gonzalez-Garibay G., Garcia-Quintana I., Pacheco-Aispuro G., Rubio C. (2024). Mechanisms of ozone-induced neurotoxicity in the development and progression of dementia: A brief review. Front. Aging Neurosci..

[26] Rodriguez P., López-Landa A., Romo-Parra H., Rubio-Osornio M., Rubio C. (2024). Unraveling the ozone impact and oxidative stress on the nervous system. Toxicology.

[27] Rubio C., Paz C. (2003). Indomethacin reverts sleep disorders produced by ozone exposure in rats. Toxicology.

[28] Brockmeyer S., D’Angiulli A. (2016). How air pollution alters brain development: The role of neuroinflammation. Transl. Neurosci..

[29] Oppenheim H.A., Lucero J., Guyot A.C., Herbert L.M., McDonald J.D., Mabondzo A., Lund A.K. (2013). Exposure to vehicle emissions results in altered blood brain barrier permeability and expression of matrix metalloproteinases and tight junction proteins in mice. Part. Fibre Toxicol..

[30] Block M.L., Calderón-Garcidueñas L. (2009). Air pollution: Mechanisms of neuroinflammation and CNS disease. Trends Neurosci..

[31] Henriquez A.R., House J.S., Snow S.J., Miller C.N., Schladweiler M.C., Fisher A., Ren H., Valdez M., Kodavanti P.R., Kodavanti U.P. (2019). Ozone-induced dysregulation of neuroendocrine axes requires adrenal-derived stress hormones. Toxicol. Sci..

[32] Daiber A., Frenis K., Kuntic M., Li H., Wolf E., Kilgallen A.B., Lecour S., Van Laake L.W., Schulz R., Hahad O. (2022). Redox regulatory changes of circadian rhythm by the environmental risk factors traffic noise and air pollution. Antioxid. Redox Signal.

[33] Mueed Z., Rai P.K., Kamal M.A., Poddar N.K. (2021). Decoding the inter-relationship between sleep disorders and Alzheimer’s disease pathogenesis. CNS Neurol. Disord. Drug Targets.

[34] Lyckenvik T., Olsson M., Forsberg M., Wasling P., Zetterberg H., Hedner J., Hanse E. (2025). Sleep reduces CSF concentrations of beta-amyloid and tau: A randomized crossover study in healthy adults. Fluids Barriers CNS.

[35] Panmanee J., Phanchana M., Pearngam P., Petchyam N., Promthep K., Wisomka P., Kutpruek S., Pannengpetch S., Prasertporn T., Mukda S. (2025). A proteomics profiling reveals the neuroprotective effects of melatonin on exogenous β-amyloid-42 induced mitochondrial impairment, intracellular β-amyloid accumulation and tau hyperphosphorylation in human SH-SY5Y cells. Cell Biol. Int..

[36] Hussain R., Graham U., Elder A., Nedergaard M. (2023). Air pollution, glymphatic impairment, and Alzheimer’s disease. Trends Neurosci..

[37] Kilburg-Basnyat B., Reece S.W., Crouch M.J., Luo B., Boone A.D., Yaeger M., Hodge M., Psaltis C., Hannan J.L., Manke J. (2018). Specialized pro-resolving lipid mediators regulate ozone-induced pulmonary and systemic inflammation. Toxicol. Sci..

[38] Smith L.C., Abramova E., Vayas K., Rodriguez J., Gelfand-Titiyevksiy B., Roepke T.A., Laskin J.D., Gow A.J., Laskin D.L. (2024). Transcriptional profiling of lung macrophages following ozone exposure in mice identifies signaling pathways regulating immunometabolic activation. Toxicol. Sci..

[39] Wu T., Li Z., Wei Y. (2023). Advances in understanding mechanisms underlying mitochondrial structure and function damage by ozone. Sci. Total Environ..

[40] Hathaway Q.A., Majumder N., Goldsmith W.T., Kunovac A., Pinti M.V., Harkema J.R., Castranova V., Hollander J.M., Hussain S. (2021). Transcriptomics of single dose and repeated carbon black and ozone inhalation co-exposure highlight progressive pulmonary mitochondrial dysfunction. Part. Fibre Toxicol..

[41] Oliveira M.M., Correia S., Peirone C., Magalhães M., Oliveira P., Peixoto F. (2024). Impact of ozone therapy on mouse liver mitochondrial function and antioxidant system. Biochimie.

[42] Ramírez-Mendoza A.A., Ramírez-Herrera M.A., Cortez-Álvarez C.R., Nery-Flores S.D., Tejeda-Martínez A.R., Romero-Prado M.M.J., Mendoza-Magaña M.L. (2022). Curcumin modifies the activity of plasmatic antioxidant enzymes and the hippocampal oxidative profile in rats upon acute and chronic exposure to ozone. Molecules.

[43] Oh C.K., Wang Y., Lipton S.A. (2025). Redox modulation of the complement cascade contributes to synapse loss in Alzheimer’s disease. Neurotherapeutics.

[44] Rivas-Arancibia S., Miranda-Martínez A., Rodríguez-Martínez E., Hernández-Orozco E., Valdés-Fuentes M., De la Rosa-Sierra R. (2023). Ozone environmental pollution: Relationship between the intestine and neurodegenerative diseases. Antioxidants.

[45] Rao Y.L., Ganaraja B., Murlimanju B.V., Joy T., Krishnamurthy A., Agrawal A. (2022). Hippocampus and its involvement in Alzheimer’s disease: A review. 3 Biotech.

[46] Sultana R., Butterfield D.A. (2010). Role of oxidative stress in the progression of Alzheimer’s disease. J. Alzheimer’s Dis..

[47] Sun Y.Y., Wang Z., Zhou H.Y., Huang H.C. (2022). Sleep-wake disorders in Alzheimer’s disease: A review. ACS Chem. Neurosci..

[48] Bello-Medina P.C., Rodríguez-Martínez E., Prado-Alcalá R.A., Rivas-Arancibia S. (2022). Ozone pollution, oxidative stress, synaptic plasticity, and neurodegeneration. Neurologia.

[49] Qu R., Sun B., Jiang J., An Z., Li J., Wu H., Wu W., Song J. (2023). Short-term ozone exposure and serum neural damage biomarkers in healthy elderly adults: Evidence from a panel study. Sci. Total Environ..

[50] Di Meco A., Joshi Y.B., Praticò D. (2014). Sleep deprivation impairs memory, tau metabolism, and synaptic integrity of a mouse model of Alzheimer’s disease with plaques and tangles. Neurobiol. Aging.

[51] Velázquez-Pérez R., Rodríguez-Martínez E., Valdés-Fuentes M., Gelista-Herrera N., Gómez-Crisóstomo N., Rivas-Arancibia S. (2021). Oxidative stress caused by ozone exposure induces changes in P2X7 receptors, neuroinflammation, and neurodegeneration in the rat hippocampus. Oxid. Med. Cell. Longev..

[52] Krueger J.M., Frank M.G., Wisor J.P., Roy S. (2016). Sleep function: Toward elucidating an enigma. Sleep Med. Rev..

[53] Liu P., Zhao B., Wei M., Li Y., Liu J., Ma L., Shang S., Huo K., Wang J., Li R. (2020). Activation of inflammation is associated with amyloid-β accumulation induced by chronic sleep restriction in rats. J. Alzheimer’s Dis..

[54] Calderón-Garcidueñas L., Reed W., Maronpot R.R., Henríquez-Roldán C., Delgado-Chavez R., Calderón-Garcidueñas A., Dragustinovis I., Franco-Lira M., Aragón-Flores M., Solt A.C. (2004). Brain inflammation and Alzheimer’s-like pathology in individuals exposed to severe air pollution. Toxicol. Pathol..

[55] Nephew B.C., Nemeth A., Hudda N., Beamer G., Mann P., Petitto J., Cali R., Febo M., Kulkarni P., Poirier G. (2020). Traffic-related particulate matter affects behavior, inflammation, and neural integrity in a developmental rodent model. Environ. Res..

[56] Rentschler K.M., Kodavanti U.P. (2024). Mechanistic insights regarding neuropsychiatric and neuropathologic impacts of air pollution. Crit. Rev. Toxicol..

[57] Roussos P., Katsel P., Fam P., Tan W., Purohit D.P., Haroutunian V. (2015). The triggering receptor expressed on myeloid cells 2 (TREM2) is associated with enhanced inflammation, neuropathological lesions and increased risk for Alzheimer’s dementia. Alzheimer’s Dement..

[58] Dean H.B., Greer R.A., Yang S.Z., Elston D.S., Brett T.J., Roberson E.D., Song Y. (2024). Multimerization of TREM2 is impaired by Alzheimer’s disease–associated variants. Alzheimer’s Dement..

[59] Greve H.J., Dunbar A.L., Lombo C.G., Ahmed C., Thang M., Messenger E.J., Mumaw C.L., Johnson J.A., Kodavanti U.P., Oblak A.L. (2023). The bidirectional lung brain-axis of amyloid-β pathology: Ozone dysregulates the peri-plaque microenvironment. Brain.

[60] Gao H.M., Zhou H., Hong J.S. (2012). NADPH oxidases: Novel therapeutic targets for neurodegenerative diseases. Trends Pharmacol. Sci..

[61] Chen S.H., Oyarzabal E.A., Hong J.S. (2016). Critical role of the Mac1/NOX2 pathway in mediating reactive microgliosis-generated chronic neuroinflammation and progressive neurodegeneration. Curr. Opin. Pharmacol..

[62] Chen S.H., Han S., Hu C.F., Zhou R., Gao Y., Tu D., Gao H., Feng J., Wang Y., Lu R.B. (2022). Activation of the MAC1-ERK1/2-NOX2 pathway is required for LPS-induced sustaining reactive microgliosis, chronic neuroinflammation and neurodegeneration. Antioxidants.

[63] Liu R.M., Chong Z., Chen J.C. (2020). Ozone and particulate matter exposure and Alzheimer’s disease: A review of human and animal studies. J. Alzheimer’s Dis..

[64] Gunawan C., Fleming C., Irga P.J., Jien Wong R., Amal R., Torpy F.R., Mojtaba Golzan S., McGrath K.C. (2024). Neurodegenerative effects of air pollutant Particles: Biological mechanisms implicated for Early-Onset Alzheimer’s disease. Environ. Int..

[65] Kang H., Si C., Shang X., Song Q., Zhang W., Li J., Jiang S. (2025). Study on the effect and mechanism of PM2.5 on the expression of Alzheimer’s disease-like pathological proteins in SH-SY5Y cells. Toxicol. Lett..

[66] Iban-Arias R., Portela A.S.D., Masieri S., Radu A., Yang E.J., Chen L.C., Gordon T., Pasinetti G.M. (2025). Role of acute exposure to environmental stressors in the gut-brain-periphery axis in the presence of cognitive resilience. Biochim. Biophys. Acta Mol. Basis Dis..

[67] Snow S.J., Broniowska K., Karoly E.D., Henriquez A.R., Phillips P.M., Ledbetter A.D., Schladweiler M.C., Miller C.N., Gordon C.J., Kodavanti U.P. (2020). Offspring susceptibility to metabolic alterations due to maternal high-fat diet and the impact of inhaled ozone used as a stressor. Sci. Rep..

[68] Zhao N., Shu Y., Jian C., Zhou Z., Bao H., Li X., Cheng X., Zhao Y., Jin S., Shu X. (2022). Lactobacillus ameliorates SD-induced stress responses and gut dysbiosis by increasing the absorption of gut-derived GABA in rhesus monkeys. Front. Immunol..

[69] Deyang T., Baig M.A.I., Dolkar P., Hediyal T.A., Rathipriya A.G., Bhaskaran M., PandiPerumal S.R., Monaghan T.M., Mahalakshmi A.M., Chidambaram S.B. (2024). Sleep apnoea, gut dysbiosis and cognitive dysfunction. FEBS J..

[70] Barrett E., Ross R.P., O’Toole P.W., Fitzgerald G.F., Stanton C. (2012). γ-Aminobutyric acid production by culturable bacteria from the human intestine. J. Appl. Microbiol..

[71] Łuc M., Misiak B., Pawłowski M., Stańczykiewicz B., Zabłocka A., Szcześniak D., Pałęga A., Rymaszewska J. (2021). Gut microbiota in dementia. Critical review of novel findings and their potential application. Prog. Neuropsychopharmacol. Biol. Psychiatry.

[72] Tranah G.J., Blackwell T., Stone K.L., Ancoli-Israel S., Paudel M.L., Ensrud K.E., Cauley J.A., Redline S., Hillier T.A., Cummings S.R. (2011). Circadian activity rhythms and risk of incident dementia and mild cognitive impairment in older women. Ann. Neurol..

[73] Uddin M.S., Tewari D., Mamun A.A., Kabir M.T., Niaz K., Wahed M.I.I., Barreto G.E., Ashraf G.M. (2020). Circadian and sleep dysfunction in Alzheimer’s disease. Ageing Res. Rev..

[74] Nguyen Ho P.T., Hoepel S.J.W., Rodriguez-Ayllon M., Luik A.I., Vernooij M.W., Neitzel J. (2024). Sleep, 24-hour activity rhythms, and subsequent amyloid-β pathology. JAMA Neurol..

[75] Huitrón-Reséndiz S., Custodio-Ramírez V., Escalante-Membrillo C., González-Piña R., Paz C. (1994). Sleep alterations and brain regional changes of serotonin and its metabolite in rats exposed to ozone. Neurosci. Lett..

[76] Paz C., Huitrón-Reséndiz S. (1996). The effects of ozone exposure on the sleep-wake cycle and serotonin contents in the pons of the rat. Neurosci. Lett..

[77] Rose M., Filiatreault A., Guénette J., Williams A., Thomson E.M. (2020). Ozone increases plasma kynurenine-tryptophan ratio and impacts hippocampal serotonin receptor and neurotrophic factor expression: Role of stress hormones. Environ. Res..

[78] Xie L., Kang H., Xu Q., Chen M.J., Liao Y., Thiyagarajan M., O’Donnell J., Christensen D.J., Nicholson C., Iliff J.J. (2013). Sleep drives metabolite clearance from the adult brain. Science.

[79] Holth J.K., Fritschi S.K., Wang C., Pedersen N.P., Cirrito J.R., Mahan T.E., Finn M.B., Manis M., Geerling J.C., Fuller P.M. (2019). The sleep-wake cycle regulates brain interstitial fluid tau in mice and CSF tau in humans. Science.

[80] Jankowska-Kieltyka M., Roman A., Nalepa I. (2021). The air we breathe: Air pollution as a prevalent proinflammatory stimulus contributing to neurodegeneration. Front. Cell. Neurosci..

[81] Schwerin S.C., Breehl N., Obasa A., Kim Y., McCabe J., Perl D.P., Haight T., Juliano S.L. (2023). Actigraphic evidence of persistent sleep disruption following repetitive mild traumatic brain injury in a gyrencephalic model. Cereb. Cortex.

[82] Calderón-Garcidueñas L., Kulesza R., Greenough G.P., García-Rojas E., Revueltas-Ficachi P., Rico-Villanueva A., Flores-Vázquez J.O., Brito-Aguilar R., Ramírez-Sánchez S., Vacaseydel-Aceves N. (2023). Fall risk, sleep behavior, and sleep-related movement disorders in young urbanites exposed to air pollution. J. Alzheimer’s Dis..

[83] Fatima G., Ashiquzzaman A., Kim S.S., Kim Y.R., Kwon H.S., Chung E. (2025). Vascular and glymphatic dysfunction as drivers of cognitive impairment in Alzheimer’s disease: Insights from computational approaches. Neurobiol. Dis..

[84] Zhang X., Jian X., Miao X., Jia Y. (2025). Notch Signaling Pathway Regulates Ozone-Induced Lung Circadian Rhythm Disruption. Toxics.

[85] Sundar I.K., Duraisamy S.K., Choudhary I., Saini Y., Silveyra P. (2023). Acute and repeated ozone exposures differentially affect circadian clock gene expression in mice. Adv. Biol..

[86] Benedusi M., Frigato E., Bertolucci C., Valacchi G. (2021). Circadian Deregulation as Possible New Player in Pollution-Induced Tissue Damage. Atmosphere.

[87] Alfaro-Rodríguez A., González-Piña R. (2005). Ozone-induced paradoxical sleep decrease is related to diminished acetylcholine levels in the medial preoptic area in rats. Chem. Biol. Interact..

[88] Rosenwasser A.M., Turek F.W. (2015). Neurobiology of circadian rhythm regulation. Sleep Med. Clin..

[89] Musiek E.S., Holtzman D.M. (2016). Mechanisms linking circadian clocks, sleep, and neurodegeneration. Science.

[90] Vriend J., Reiter R.J. (2015). Melatonin feedback on clock genes: A theory involving the proteasome. J. Pineal Res..

[91] Cardinali D.P. (2019). Are melatonin doses employed clinically adequate for melatonin-induced cytoprotection?. Melatonin Res..

[92] Sevastre-Berghian A.C., Casandra C., Gheban D., Olteanu D., Olanescu Vaida Voevod M.C., Rogojan L., Filip G.A., Bâldea I. (2022). Neurotoxicity of bisphenol A and the impact of melatonin administration on oxidative stress, ERK/NF-kB signaling pathway, and behavior in rats. Neurotox Res..

[93] Mizuno T., Higo S., Kamei N., Mori K., Sakamoto A., Ozawa H. (2022). Effects of general anesthesia on behavioral circadian rhythms and clock-gene expression in the suprachiasmatic nucleus in rats. Histochem. Cell Biol..

[94] Albadawi E.A. (2025). Structural and functional changes in the hippocampus induced by environmental exposures. Neurosciences.

[95] Scassellati C., Galoforo A.C., Esposito C., Ciani M., Ricevuti G., Bonvicini C. (2021). Promising intervention approaches to potentially resolve neuroinflammation and steroid hormones alterations in Alzheimer’s disease and its neuropsychiatric symptoms. Aging Dis..

[96] Calderón-Garcidueñas L., Cejudo-Ruiz F.R., Stommel E.W., González-Maciel A., Reynoso-Robles R., Silva-Pereyra H.G., Pérez-Guille B.E., Soriano-Rosales R.E., Torres-Jardón R. (2025). Sleep and arousal hubs and ferromagnetic ultrafine particulate matter and nanoparticle motion under electromagnetic fields: Neurodegeneration, sleep disorders, orexinergic neurons, and air pollution in young urbanites. Toxics.

[97] Tang M., Li D., Liew Z., Wei F., Wang J., Jin M., Chen K., Ritz B. (2020). The association of short-term effects of air pollution and sleep disorders among elderly residents in China. Sci. Total Environ..

[98] Christiansen A., Mickley L.J., Liu J., Oman L.D., Hu L. (2022). Multidecadal increases in global tropospheric ozone derived from ozonesonde and surface site observations: Can models reproduce ozone trends?. Atmos. Chem. Phys..

[99] Yan Y.N., Williams J.P., Niu K., Zhang W.H., Zhang J.F., Shi L., An J.X. (2022). Intraperitoneal ozone injection prevents REM sleep deprivation-induced spatial learning and memory deficits by suppressing the expression of Sema3A in the hippocampus in rats. Iran J. Basic Med. Sci..

[100] Cleary E.G., Cifuentes M., Grinstein G., Brugge D., Shea T.B. (2018). Association of low-level ozone with cognitive decline in older adults. J. Alzheimer’s Dis..

[101] Lo K., Chiang L.L., Hsu S.M., Tsai C.Y., Wu D., Chou C.J., Chuang H.C., Liu W.T. (2021). Association of short-term exposure to air pollution with depression in patients with sleep-related breathing disorders. Sci. Total Environ..

[102] Meo S.A., Shaikh N., Alotaibi M., AlWabel A.A., Alqumaidi H. (2024). Effect of air pollutants particulate matter (PM2.5, PM10), sulfur dioxide (SO_2_) and ozone (O_3_) on cognitive health. Sci. Rep..

[103] Bhuniya S., Goyal M., Chowdhury N., Mishra P. (2022). Intermittent hypoxia and sleep disruption in obstructive sleep apnea increase serum tau and amyloid-beta levels. J. Sleep Res..

[104] Tsai C.Y., Su C.L., Huang H.T., Lin H.W., Lin J.W., Hei N.C., Cheng W.H., Chen Y.L., Majumdar A., Kang J.H. (2025). Mediating role of obstructive sleep apnea in altering slow-wave activity and elevating Alzheimer’s disease risk: Pilot study from a northern Taiwan cohort. Sleep Health.

[105] Trelle A.N., Young C.B., Vossler H., Ramos Benitez J., Cody K.A., Mendiola J.H., Swarovski M.S., Guen Y.L., Feinstein I., Butler R.R. (2025). Plasma Aβ42/Aβ40 is sensitive to early cerebral amyloid accumulation and predicts risk of cognitive decline across the Alzheimer’s disease spectrum. Alzheimer’s Dement..

[106] US Environmental Protection Agency (EPA) (2015). National Ambient Air Quality Standards for Ozone; Final Rule. Fed Regist..

[107] Re L., Martínez-Sánchez G., Bordicchia M., Malcangi G., Pocognoli A., Morales-Segura M.A., Rothchild J., Rojas A. (2014). Is ozone pre-conditioning effect linked to Nrf2/EpRE activation pathway in vivo? A preliminary result. Eur. J. Pharmacol..

[108] Tricarico G., Travagli V. (2021). The Relationship between Ozone and Human Blood in the Course of a Well-Controlled, Mild, and Transitory Oxidative Eustress. Antioxidants.

[109] Shehata N.I., Abd-Elgawad H.M., Mawsouf M.N., Shaheen A.A. (2012). The potential role of ozone in ameliorating the age-related biochemical changes in male rat cerebral cortex. Biogerontology.

[110] Resitoglu B., Celik Y., Komur M., Polat A., Erdogan S., Arslankoylu A.E., Beydagi H. (2018). The efficacy of ozone therapy in neonatal rats with hypoxic ischemic brain injury. Bratisl. Lek Listy..

[111] Zhu L., Ding S., Xu L., Wu Z. (2022). Ozone treatment alleviates brain injury in cerebral ischemic rats by inhibiting the NF-κB signaling pathway and autophagy. Cell Cycle.

[112] Bjørklund G., Gurgas L., Hangan T. (2025). Ozone-induced neurotoxicity: Mechanistic insights and implications for neurodegenerative diseases. Curr. Med. Chem..

[113] Zhang X., Wang S.J., Wan S.C., Li X., Chen G. (2025). Ozone: Complicated effects in central nervous system diseases. Med. Gas Res..

